# Predictors of left ventricular ejection fraction in high-risk percutaneous coronary interventions

**DOI:** 10.3389/fcvm.2024.1342409

**Published:** 2024-02-02

**Authors:** Vasileios F. Panoulas, Javier Escaned, Jonathan M. Hill, Erin Barker, Karin Butler, Ali Almedhychy, Stelios I. Tsintzos, William W. O’Neill

**Affiliations:** ^1^ Department of Cardiology, Harefield Hospital, Royal Brompton and Harefield Hospitals, Guy’s and St Thomas’ NHS Foundation Trust, London, United Kingdom; ^2^ Department of Interventional Cardiology, Hospital Clinico San Carlos, Madrid, Spain; ^3^ Department of Cardiology, Royal Brompton Hospital, Royal Brompton and Harefield Hospitals, Guy's and St Thomas' NHS Foundation Trust, London, United Kingdom; ^4^ York Health Economics Consortium, University of York, York, United Kingdom; ^5^ Medical Affairs, Abiomed Inc., Danvers, MA, United States; ^6^ Health Economics and Market Access, Abiomed Europe GmbH, Aachen, Germany; ^7^ Centre for Structural Heart Disease, Henry Ford Hospital, Detroit, MI, United States

**Keywords:** revascularization completeness, residual SYNTAX score, left ventricular ejection fraction, percutaneous coronary intervention, intra-aortic balloon pump, micro-axial heart pump, percutaneous ventricular assist device, Impella

## Abstract

Revascularization completeness after percutaneous coronary intervention (PCI) is associated with improved long-term outcomes. Mechanical circulatory support [intra-aortic balloon pump (IABP) or Impella] is used during high-risk PCI (HR-PCI) to enhance peri-procedural safety and achieve more complete revascularization. The relationship between revascularization completeness [post-PCI residual SYNTAX Score (rSS)] and left ventricular ejection fraction (LVEF) in HR-PCI has not been established. We investigated LVEF predictors at 90 days post-PCI with Impella or IABP support. Individual patient data (IPD) were analyzed from PROTECT II (NCT00562016) in the base case. IPD from PROTECT II and RESTORE-EF (NCT04648306) were naïvely pooled in the sensitivity analysis. Using complete cases only, linear regression was used to explore the predictors of LVEF at 90 days post-PCI. Models were refined using stepwise selection based on Akaike Information Criterion and included: treatment group (Impella, IABP), baseline characteristics [age, gender, race, New York Heart Association Functional Classification, LVEF, SYNTAX Score (SS)], and rSS. Impella treatment and higher baseline LVEF were significant predictors of LVEF improvement at 90 days post-PCI (*p* ≤ 0.05), and a lower rSS contributed to the model (*p* = 0.082). In the sensitivity analysis, Impella treatment, higher baseline LVEF, and lower rSS were significant predictors of LVEF improvement at 90 days (*p* ≤ 0.05), and SS pre-PCI contributed to the model (*p* = 0.070). Higher baseline LVEF, higher SS pre-PCI, lower rSS (i.e. completeness of revascularization), and Impella treatment were predictors of post-PCI LVEF improvement. The findings suggest potential mechanisms of Impella include improving the extent and quality of revascularization, and intraprocedural ventricular unloading.

## 1 Introduction

In stable coronary artery disease (CAD), revascularization has been shown to increase patient survival and reduce rehospitalization (1). The two primary methods of revascularization are coronary artery bypass grafting (CABG) and percutaneous coronary intervention (PCI). In patients with severe left ventricular systolic impairment, there is an ongoing debate as to whether PCI with the use of mechanical circulatory support (MCS) could be a safer alternative to CABG with a wider population applicability (2).

A substantial number of PCI procedures are performed globally each year (3), with an increasing proportion of these patients classed as high-risk PCI (HR-PCI). This is due to advancing age, increasing comorbidities, and more complex disease, alongside the higher prevalence of CAD (4). These patients are likely to be deemed ineligible for CABG surgery. While a standardized definition of HR-PCI remains unclear, several criteria are considered including clinical, anatomical and hemodynamic factors (5, 6).

Clinical guidelines (from the UK, US and Europe) have included completeness of revascularization as a key component of successful PCI (7–9). These also recommend, where possible, one-stage index revascularization over multi-stage procedures, as this is thought to improve clinical outcomes and be cost saving (8, 10).

MCS devices have been used during HR-PCI procedures to achieve more complete revascularization and enhance safety by maintaining hemodynamic stability and tissue perfusion throughout the procedure (11). The intra-aortic balloon pump (IABP) was previously the primarily used MCS device that can increase cardiac output by 0.5–1 L/min (12). Impella is a small micro-axial flow heart pump that pumps a maximum flow of 2.5 L/min and 4.3 L/min for the Impella 2.5 and Impella CP with SmartAssist models, respectively (13, 14).

PROTECT II compared patient outcomes after HR-PCI with Impella 2.5 vs. IABP support. The Impella device was associated with improved hemodynamic support and significantly lower rates of major adverse events at 90 days compared with the IABP (15). RESTORE-EF observed that HR-PCI with Impella led to significantly improved left ventricular ejection fraction (LVEF) at 90 days (16). LVEF is an important predictor of long-term outcomes (17). It was reported previously that for patients with an LVEF of less than 40%, mortality risk increases by 26% for every 5% decrease in LVEF (18).

The objective of this study was to explore the predictor variables for LVEF at 90 days in patients undergoing HR-PCI with either Impella or IABP support. This has not been previously investigated in HR-PCI or other populations.

## 2 Method

In this study, the predictors of LVEF at 90 days post-PCI (with Impella or IABP support) were investigated using linear regression analysis.

### 2.1 Patient selection

Individual patient data from PROTECT II (NCT00562016) and RESTORE-EF (NCT04648306) were analyzed. The base case analysis was conducted using data from the PROTECT II randomized controlled trial. The base case analysis refers to the analysis of randomized controlled trial data, using the most likely or preferred set of assumptions and input values.

The sensitivity analysis was performed to maximize the amount of available data and was used to assess the robustness of the statistical model. The sensitivity analysis was a naïve pooled analysis of data from PROTECT II and RESTORE-EF. Further details of PROTECT II and RESTORE-EF were reported previously (19, 20).

All statistical analyses included complete cases only. Complete cases were defined as those patients with no missing data for all baseline characteristics or outcome variables of interest. It was assumed that any missing data were missing completely at random. Patients with any missing data were excluded from this analysis. Supplementary Figures S1, S2 provide further detail on how patients were included and excluded from the analysis.

### 2.2 Statistical analysis

A *t*-test and chi-squared test for continuous and categorical data, respectively, were used to identify any differences in baseline characteristics between treatment groups (Impella or IABP) for the base case and sensitivity analyses.

Linear regression analysis was performed in R (version 4.2.1) to explore the relationship between LVEF (at 90 days) and the following independent variables:

• Treatment group (Impella or IABP)
• Baseline age
• Gender
• Race
• Baseline New York Heart Association (NYHA) functional classification (21)
• Baseline LVEF (%)
• Baseline SYNTAX score (SS)
• Residual SYNTAX score (rSS), used to quantify the extent of revascularization (22)

All regression models were refined using stepwise selection based on Akaike Information Criterion (AIC). Lower AIC values can indicate a more parsimonious model; therefore, the model with the lowest AIC was considered the preferred model. Stepwise selection was performed using the “stepAIC” function in R. Since the preferred model is based on AIC values, the coefficient and significance of the predictor variables that remain in the model should be reviewed and understood. All variables with a *p*-value ≤ 0.05 were considered statistically significant though the statistically “best” fitting model may include non-significant variables.

## 3 Results

### 3.1 Baseline characteristics

Out of 448 patients in PROTECT II, 75% had available NYHA functional classification data, 67% had available SS pre-PCI data, and 98% had available baseline LVEF data. Post-procedural LVEF data at 90 days were available for 71% of patients. Therefore, 165 complete cases from PROTECT II were included in the base case analysis. Similarly, out of 406 patients in RESTORE-EF, 75% had available NYHA data, 76% had available SS pre-PCI data, and >99% had available baseline LVEF data. Post-procedural LVEF data were available for 72% of patients at 90 days resulting in 182 RESTORE-EF complete cases included in the sensitivity analysis.

The baseline characteristics of the IABP and Impella groups from PROTECT II for the base case analysis and of the IABP (from PROTECT II) and Impella (from PROTECT II and RESTORE-EF) groups for the sensitivity analysis are shown in Table 1.

**Table 1:** Baseline characteristics.

Baseline characteristics	Base case analysis			Sensitivity analysis		
	Impella (*n* = 88)	IABP (*n* = 77)	*p*-value	Impella (*n* = 270)	IABP (*n* = 77)	*p*-value
Age (years), mean (SD)	67.22 (11.58)	67.26 (10.68)	0.98	69.34 (11.73)	67.26 (10.68)	0.16
Males, *n* (%)	72 (81.80)	62 (80.50)	0.99	214 (79.30)	62 (80.50)	0.94
Race, *n* (%)			0.87			0.38
American Indian or Alaska native	1 (1)	0 (0)		1 (0.40)	0 (0)	
Asian	3 (3.40)	3 (3.90)		6 (2.20)	3 (3.90)	
Black or African American	13 (14.80)	13 (16.90)		26 (9.60)	13 (16.90)	
Native Hawaiian or other Pacific Islander	1 (1.10)	1 (1.30)		1 (0.40)	1 (1.30)	
Other	4 (4.50)	6 (7.80)		25 (9.30)	6 (7.80)	
White	66 (75.00)	54 (70.10)		211 (78.10)	54 (70.10)	
NYHA class, *n* (%)			0.84			0.02*
NYHA class I	5 (5.70)	4 (5.20)		20 (7.40)	4 (5.20)	
NYHA class II	24 (27.30)	26 (33.80)		57 (21.10)	26 (33.80)	
NYHA class III	42 (47.70)	34 (44.20)		105 (38.90)	34 (44.20)	
NYHA class IV	17 (19.30)	13 (16.90)		88 (32.60)	13 (16.90)	
LVEF (%), mean (SD)	24.53 (7.27)	24.92 (8.16)	0.75	31.60 (14.43)	24.92 (8.16)	<0.001*
SS pre-PCI, mean (SD)	29.17 (12.99)	27.80 (12.84)	0.50	30.23 (14.11)	27.80 (12.84)	0.18

IABP, intra-aortic balloon pump; LVEF, left ventricular ejection fraction; NYHA, New York Heart Association Functional Classification; SD, standard deviation; SS, SYNTAX Score.

* Significant at the *p* ≤ 0.05 significance level.

There are no statistically significant differences between treatment groups (Impella vs. IABP) in the baseline characteristics for the base case analysis. However, there is an imbalance in the baseline characteristics between treatment groups in the sensitivity analysis, with statistically significant differences (*p* ≤ 0.05) for baseline LVEF and NYHA classification. Patients treated with IABP had a lower baseline LVEF compared with patients treated with Impella. Furthermore, there was a lower proportion of NYHA Class I and Class IV patients and a higher proportion of Class II and Class III patients in the IABP group compared with the Impella group.

### 3.2 Post-procedural characteristics

The post-procedural characteristics of the IABP and Impella groups from PROTECT II for the base case analysis and of the IABP (from PROTECT II) and Impella (from PROTECT II and RESTORE-EF) groups for the sensitivity analysis are shown in Table 2. Treatment with Impella, compared with IABP, was associated with an increase in absolute LVEF, which can be seen in a numerical, but not significant, advantage at 90 days in the base case analysis.

**Table 2:** 90-day post-PCI characteristics.

Post-procedural characteristics	Base case analysis			Sensitivity analysis		
	Impella (*n* = 88)	IABP (*n* = 77)	*p*-value	Impella (*n* = 270)	IABP (*n* = 77)	*p*-value
LVEF (%), mean (SD)	32.49 (12.02)	30.00 (11.07)	0.17	40.93 (14.99)	30.00 (11.07)	<0.001*
Average change in LVEF (%), median (IQR)	10.00 (0.00, 15.00)	5.00 (0.00, 10.00)	0.025*	9.00 (0.00, 15.00)	5.00 (0.00, 10.00)	0.003*
rSS, mean (SD)	13.71 (12.02)	13.60 (12.42)	0.956	8.24 (11.53)	13.60 (12.42)	<0.001*
NYHA class, *n* (%)			0.482			0.128
NYHA class I	40 (50.00)	31 (48.40)		123 (56.70)	31 (48.40)	
NYHA class II	22 (27.50)	18 (28.10)		49 (22.60)	18 (28.10)	
NYHA class III	16 (20.00)	10 (15.60)		40 (18.40)	10 (15.60)	
NYHA class IV	2 (2.50)	5 (7.80)		5 (2.30)	5 (7.80)	

IABP, intra-aortic balloon pump; LVEF, left ventricular ejection fraction; NYHA, New York Heart Association Functional Classification; PCI, percutaneous coronary intervention; rSS, residual SYNTAX Score; SD, standard deviation.

* Significant at the *p* ≤ 0.05 significance level.

### 3.3 Base case analysis

The base case analysis of LVEF at 90 days included 165 patients (88 Impella; 77 IABP) from the PROTECT II trial (Table 3). Treatment group and baseline LVEF remained in the model as significant predictors of LVEF at 90 days post-PCI (both *p* ≤ 0.05). Additionally, rSS had a numerical but non-significant contribution to the model (*p* = 0.082).

**Table 3:** Base case analysis: predictors of LVEF at 90 days post-PCI.

Variable	Coefficient	SE	*p*-value
Treatment group (Impella)	2.85	1.44	0.050
LVEF at baseline	0.89	0.09	0.000*
rSS	−0.10	0.06	0.082

LVEF, left ventricular ejection fraction; PCI, percutaneous coronary intervention; rSS, residual SYNTAX Score; SE, standard error.

* Significant at the *p* ≤ 0.05 significance level.

Treatment with Impella, compared with IABP, was associated with a 2.85% increase in absolute LVEF at 90 days. The average increase in LVEF from baseline to 90-day post-PCI with Impella was significantly higher than the average increase with IABP (10% median LVEF increase with Impella vs. 5% median increase with IABP, *p* = 0.025). A one-unit (%) increase in baseline LVEF was associated with a 0.89% increase in absolute LVEF (%) at 90 days. A one-unit increase in rSS was associated with a 0.10% decrease in absolute LVEF (%) at 90 days.

### 3.4 Sensitivity analysis

The sensitivity analysis included 347 patients (270 Impella; 77 IABP) from PROTECT II and RESTORE-EF (Table 4). Treatment group, baseline LVEF, and rSS remained in the model as significant predictors of LVEF at 90 days post-PCI (all *p* ≤ 0.05). Additionally, SS pre-PCI had a numerical but non-significant contribution to the model (*p* = 0.070).

**Table 4:** Sensitivity analysis: predictors of LVEF at 90 days post-PCI.

Variable	Coefficient	SE	*p*-value
Treatment group (Impella)	4.76	1.42	0.001*
LVEF at baseline	0.67	0.04	<0.001*
SS pre-PCI	0.09	0.05	0.070
rSS	−0.27	0.06	<0.001*

LVEF, left ventricular ejection fraction; PCI, percutaneous coronary intervention; rSS, residual SYNTAX Score; SE, standard error; SS, SYNTAX Score.

* Significant at the *p* ≤ 0.05 significance level.

Treatment with Impella, compared with IABP, was associated with a 4.75% increase in absolute LVEF (%) at 90 days. The average increase in LVEF from baseline to 90-day post-PCI with Impella was significantly higher than the average increase with IABP (9% median LVEF increase with Impella vs. 5% median increase with IABP, *p* = 0.003). A one-unit (%) increase in baseline LVEF was associated with a 0.67% increase in absolute LVEF (%) at 90 days. A one-unit increase in rSS was associated with a 0.27% decrease in absolute LVEF (%) at 90 days. A one-unit increase in SS pre-PCI was associated with a 0.09% increase in absolute LVEF (%) at 90 days.

## 4 Discussion

In the current study we demonstrated that in patients with stable complex CAD and severe left ventricular impairment, a higher baseline LVEF, a lower rSS, and treatment with Impella are independent predictors of LVEF improvement at 90 days post-PCI.

The present analysis showed that LVEF at baseline was a significant predictor of LVEF post-PCI at 90 days. This is somewhat expected as baseline health is likely to influence this outcome, and previous studies have identified baseline LVEF as an important predictor of left ventricular function after PCI (23).

Another important finding of this work was that rSS (i.e., completeness of revascularization) was a predictor of LVEF at 90 days. This finding was only significant in the sensitivity analysis, where more patients achieved complete revascularization (rSS = 0) and rSS <8. This could be attributed to the higher level of experience in the centers contributing patients to the sensitivity analysis, together with enhanced procedural practices and technically advanced versions of Impella (RESTORE-EF used predominately Impella CP with SmartAssist, an enhancement to the Impella 2.5 used in PROTECT II) leading to more optimal revascularization. Other studies have also shown more complete revascularization to be associated with significant improvement of LVEF (24). Similar investigations exploring the predictors of clinical outcomes have been undertaken in acute myocardial infarction and following PCI in patients with stable CAD (25, 26). However, this is the first study, to our knowledge, to show a direct, quantifiable relationship between rSS and LVEF. Previous work has also reported that patients with rSS ≤8 had comparable long-term mortality to those with complete revascularization, whereas rSS >8 were associated with increasing adverse long-term clinical outcomes (22). Future randomized studies should be undertaken to explore the relationships with LVEF in these subgroups.

Treatment with Impella was a significant predictor of LVEF at 90 days in the base case and sensitivity analyses. Potential mechanisms of LVEF improvement in HR-PCI patients treated with Impella include completeness of revascularization, quality of revascularization, and intraprocedural ventricular unloading which could minimize periprocedural myocardial injury.

Previous studies have identified the importance of the extent and quality of revascularization in PCI, and the necessity of using decalcification techniques such as atherectomy or intracoronary lithotripsy to tackle the hardest of lesions (27). In the current study, we also demonstrate that Impella use is a significant predictor of LVEF improvement independent of rSS. This could suggest that quality of revascularization plays an equally important role on future LVEF recovery.

Interestingly, SS pre-PCI did not remain in the base case model and had a numerical but non-significant contribution to the sensitivity analysis model. It was expected that more complex disease at baseline would be significantly associated with greater improvements in LVEF post-PCI, due to these patients having “more to gain” from revascularization. Therefore, further research is needed to determine the extent to which SS pre-PCI predicts outcomes at 90 days. It is important to note that in both data sets, baseline SS indicated substantial anatomical complexity. Had the analysis included patients with less complex coronary lesions, the regression model might have arrived at different results.

### 4.1 Limitations

Several limitations were identified in the present study. Firstly, randomized controlled trial data were combined with observational data from RESTORE-EF for the sensitivity analysis. There were significant differences in the baseline characteristics between treatment groups in the pooled sensitivity analysis, meaning these results should be interpreted with caution. There are also limitations associated with using observational data in general, such as selection bias (28). While the base case analysis was based on PROTECT II data only, which was considered the most robust, this was potentially underpowered, so future investigations should investigate the relationships with a larger sample size.

Furthermore, all missing data were assumed to be missing completely at random. Reasons for missing data in PROTECT II and RESTORE-EF could be due to the difficulty of calculating rSS and LVEF and/or that centers were not required or incentivized to collect this information. It is possible that the patients with missing data who were excluded from the analyses differed to those who were included. Missing data in trial sets is common and is expected, to some extent, even where efforts are made to minimize data loss. Complete case analysis reduces the overall number of data points available. In this analysis, it was necessary that a patient had complete data for all variables and outcomes of interest. As our analysis sought to understand the influence of baseline variables on outcomes, we preferred not to impute data to avoid drawing conclusions about relationships that were already subject to predictive algorithms and necessary assumptions. Additionally, all the analyzed IABP data were included from a single source which could increase bias. Although this is a potential limitation, additional high-quality IABP data sources were unavailable.

Another important factor is that the Impella 2.5 device was used in the PROTECT II trial. However, the Impella CP with SmartAssist is the newer device of choice for HR-PCI cases and is increasingly being used in research studies (20, 29). Given that the peak flow rate with the Impella CP with SmartAssist is 4.3 L/min (vs. 2.5 L/min for Impella 2.5 (13), it is plausible that the relationships observed in this study could be enhanced with the newer device. Further research should be conducted to explore this. An additional randomized controlled trial, PROTECT IV (NCT04763200), is expected to be complete in 2027 and is hoped to provide further insight into Impella during HR-PCI compared with IABP.

## 5 Conclusion

The current study demonstrates that in patients with stable complex CAD and severe left ventricular impairment, a higher baseline LVEF, a lower rSS (i.e., completeness of revascularization), and treatment with Impella are independent predictors of LVEF improvement at 90 days post-PCI. The findings suggest that potential mechanisms of Impella could include improving the extent and quality of revascularization, and intraprocedural ventricular unloading. However, the sensitivity analyses were based on data from a randomized and an observational study, hence should be reviewed with caution. Ongoing randomized controlled trials using Impella CP with SmartAssist will provide further insight in the role of Impella during HC-PCI in improving LVEF and outcomes compared to current standard of care.

## Data Availability

The data analyzed in this study is subject to the following licenses/restrictions: the datasets presented in this article are not readily available because these are commercial studies that need to be treated as confidential. Requests to access these datasets should be directed to VP, v.panoulas@rbht.nhs.uk.
