# Supplementary material for: Predictors of left ventricular ejection fraction in high-risk percutaneous coronary interventions
Source: Front Cardiovasc Med. 2024 Feb 2;11:1342409. doi: 10.3389/fcvm.2024.1342409 (PMC10869567; doi:10.3389/fcvm.2024.1342409)
Supplement: Supplementary file 1 [file Datasheet1.docx]

Supplementary Material

## Supplementary Figures


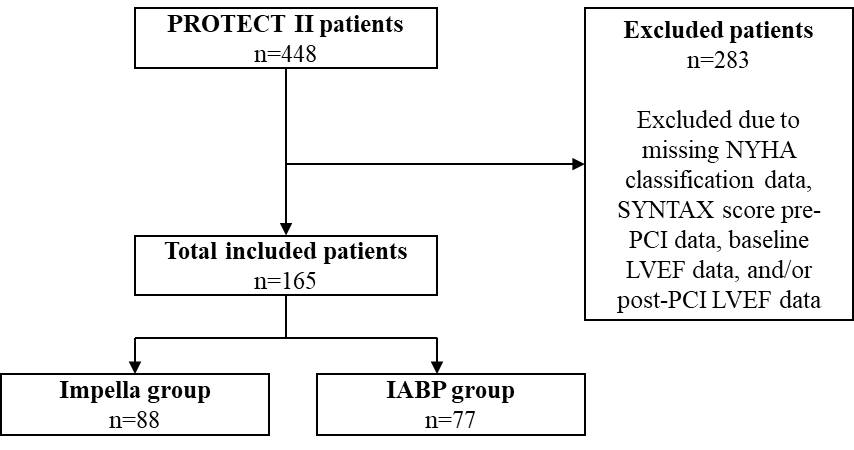


Key: IABP – intra-aortic balloon pump, LVEF – left ventricular ejection fraction, NYHA – New York Heart Association, PCI – percutaneous coronary intervention.

**Supplementary Figure 1.** A flowchart showing the number of PROTECT II patients included in the base case analysis.

**
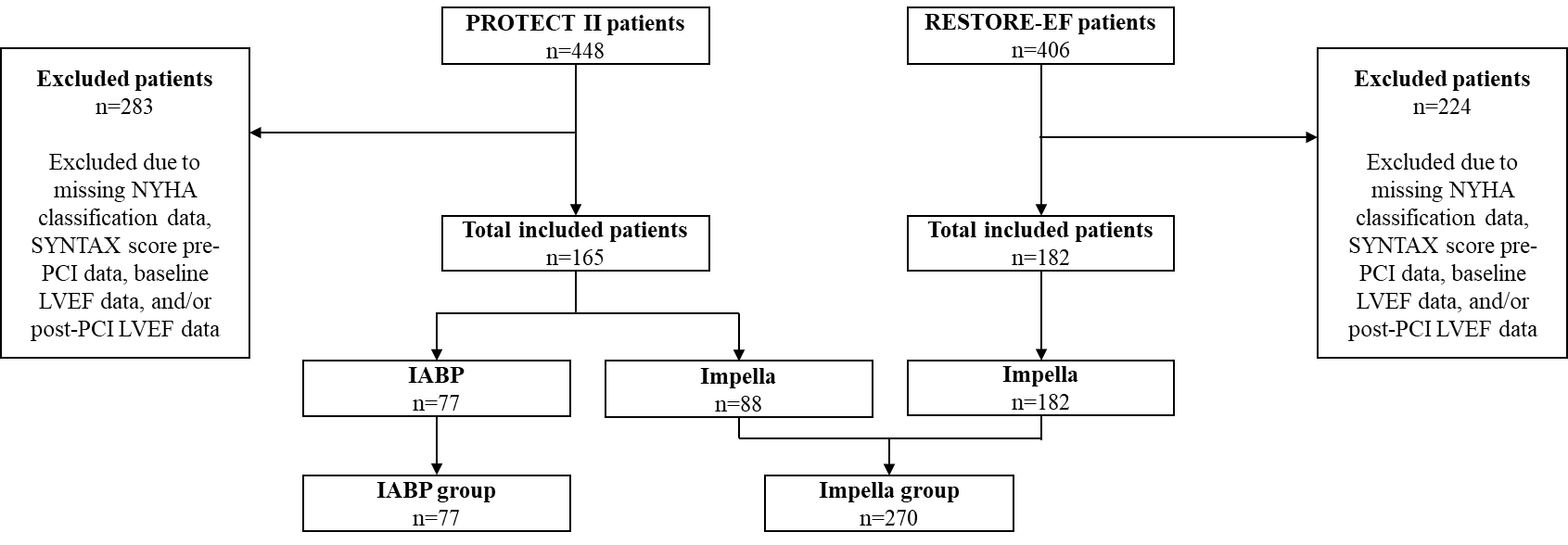
**

Key: IABP – intra-aortic balloon pump, LVEF – left ventricular ejection fraction, NYHA – New York Heart Association, PCI – percutaneous coronary intervention.

**Supplementary Figure 2.** A flowchart showing the number of PROTECT II and RESTORE-EF patients included in the sensitivity analysis.
